# Medical Societies in the United States: History, Purpose, Challenge, and Future Directions

**DOI:** 10.7759/cureus.112407

**Published:** 2026-07-10

**Authors:** Gary Schiller, Leo R Leer

**Affiliations:** 1 Hematology and Oncology, David Geffen School of Medicine at UCLA, Los Angeles, USA; 2 Family Medicine, Providence Medical Group Eureka, Eureka, USA

**Keywords:** clinical relevance, leadership, medical societies, policy, social advocacy

## Abstract

Medical specialty and subspecialty societies were established to represent the interests of their profession, with both scientific and political objectives. Medical societies have long served as central institutions within the U.S. medical profession, developing standards of practice, supporting education, and advancing research. Many also take their potential role in advancing access and shaping public opinion of the profession seriously. In this manuscript, we discuss the history of these societies in the United States, their evolution, and the opportunities that exist to make medical societies more relevant to the needs of current physicians.

## Editorial

Introduction

The first organized medical societies in the United States emerged in the late 18th and early 19th centuries, often at state and local levels. These organizations followed models of royal societies or guilds that were established in Europe to strengthen the then-new scientific underpinnings of medicine and to advance the often-political position of medical science [1]. The Massachusetts Medical Society, founded in 1781, and the New York State Medical Society, founded in 1807, were among the earliest on this continent. Like their European counterparts, these organizations were formed to establish professional standards, combat quackery, and legitimize medicine as a learned profession [1].

The American Medical Association (AMA), established in 1847, represented a pivotal moment in attempting to unify physicians nationally [1]. For the first century of its existence, the AMA created frameworks for medical ethics, standardized medical education, and advocated for scientific rigor [1]. Over time, as the scope of medical knowledge expanded, societies focused on specialties and subspecialties (e.g., American College of Physicians, American Society of Hematology, American Society of Clinical Oncology, etc.) were established, reflecting the increasing complexity and fragmentation of medicine.

Subsequently, medical societies expanded their political and social advocacy. The AMA, for example, campaigned vigorously to protect the economic interests of physicians, especially through the 1940s and 1950s, when it played on McCarthy-era fears of socialism to help defeat both President Truman’s and President Eisenhower’s efforts to enact national health insurance [1].

At its peak in the mid-1960’s, nearly 70% of U.S. physicians were AMA members. Since then, membership has steadily declined, so that since 2010, fewer than 25% of U.S. physicians have joined the AMA [2]. Today, many physicians instead belong exclusively to their specialty societies. For example, roughly 68% of practicing family physicians belong to the American Academy of Family Physicians (AAFP), and roughly 70-90% of practicing U.S. oncologists belong to the American Society of Clinical Oncologists (ASCO) [2]. Why do physicians find their specialty societies to be more relevant to their daily needs? Given the history of physician involvement with the AMA as a cautionary tale, we should also ask if our specialty societies are evolving rapidly enough to maintain their relevance. In this era when most physicians are essentially employed by medium to large or very large health care organizations, what role can specialty societies play in improving the professional lives of physicians? Finally, what can our societies do to maintain and increase their appeal to the younger generation of physicians [3]?

As noted, medical societies have long served as central institutions within the U.S. medical profession, developing standards of practice, supporting education, and advancing research. Many also take their potential role in advancing access and shaping public opinion of the profession seriously [4,5]. As medicine has grown increasingly specialized, so too have professional societies, offering clinicians, researchers, and educators forums for collaboration, leadership development, and advocacy. This manuscript examines the current state of medical societies, their functions for different specialties, the nature and development of leadership within them, and the challenges they face in the modern era. Finally, we will propose recommendations for how medical societies might remain relevant to future generations of physicians.

Purposes of medical societies

Medical societies today serve multiple overlapping purposes that include developing professional identity and providing physicians a sense of belonging to a professional community beyond institutional boundaries. Since the mid-20th century, societies have, to a varying degree, stressed scientific advancement and achievement through annual meetings and exhibitions, as well as the publication of journals. Scientific gatherings and the proliferation of publications have also served as major sources of revenue of these medical societies.

Another major area of scientific and clinical content consists of developing standards and guidelines. Societies often lead in defining best practices, diagnostic criteria, and treatment algorithms in their respective fields. These ventures provide a major avenue for peer education, but less so for education of the public at large [4].

More recently, leaders in many subspecialty societies have developed programs addressing national challenges in education and training that include continuing medical education (CME), mentorship programs, and dissemination of clinical consensus guidelines [6]. Moreover, the AAFP has a host of “toolboxes” that physicians can tap into, including help with personal and professional finances and discount programs on clinical and non-clinical services, as well as less traditional training modules, such as recognizing implicit bias and strategies for reaching underserved populations [7]. Furthermore, recognizing that many young physicians are dissatisfied with corporate employment options that are increasingly becoming the norm, the AAFP offers services aimed at helping physicians develop direct primary care practices.

These varied services have, by necessity, led to the recruitment of a professional staff of highly trained career personnel. This cohort of non-physician professionals typically serves as institutional memory for the organizations, although developing and monitoring society offerings typically remains vested in the societies’ volunteer physician leadership.

As in the past, many, if not all, medical societies in the United States continue their efforts at political advocacy. Advocacy and positions are designed to represent the interests of the membership in healthcare policy, research funding, and regulatory environments, although how the interests of the membership are assessed varies greatly among these organizations. Some societies have recognized the value of involving community activists on committees and on advisory boards, but the breadth and depth of patient involvement remain limited.

Leadership in medical societies: development and definition

Leadership within medical societies consists of both formal and informal positions. Formal leadership structures are dictated by the rules required for tax-exempt status wherever they are incorporated. Most societies are governed by elected officers (president, vice president, and board members). In subspecialty societies, these leaders are often drawn from academic or clinical roles historically recognized for their scholarship, service, or medical innovation. In the AAFP, leaders may come from actively practicing, non-academic ranks; however, for most primary care physicians, taking on the burden of a time-consuming, largely volunteer job makes involvement difficult.

Pathways to leadership typically include committee service, editorial boards, mentorship roles, and program development. Leadership often evolves through active participation in society initiatives, demonstrating commitment to the mission of the organization. At times, recruitment for leadership follows those individuals who have been recruited to leadership in parallel societies, limiting access to those with a history of administrative service. The result is that, for many similar societies, leadership largely comes from a small pool of individuals who rotate from one society to the next rather than being developed from within the grassroots of each society [8].

Challenges facing the medical profession, research, and education

Several pressing challenges threaten the vitality of medical societies. Professionally, physician burnout, declining autonomy, and shifting healthcare economics require societies to advocate for physician well-being and sustainable practice models. How well they carry out initiatives to support the profession has not been measured. We are not, for example, aware of any data suggesting that medical societies play any role in reducing physician burn-out [9]. Societies may make recommendations for interventions through government-action committees that identify sources of burnout, such as the electronic medical record, documentation and billing, and the declining role of physicians in healthcare administration. Societies can be charged with developing a tool-box to identify features of physician burnout and interventions specific to the membership.

We must develop metrics to measure how effective societies are at reducing such burdens; for example, can societies effectively lobby for reduction of the growing non-clinical paperwork that flourishes in healthcare systems, and can lobbying efforts be measured? If physician organizations cannot keep up with the demands of systems, young physicians will jettison membership and drop attending scientific meetings. Indeed, one author can attest anecdotally to the near-total lack of family physician interest in the AAFP in his region.

This may result because young physicians in practice are almost all working within health systems that provide and control (for better or worse) all their professional and financial needs. They have little time or interest to give to a distant organization with which they interact (rarely) via a web interface. Moreover, many young primary care physicians may express more interest in using their employer-provided CME stipend to attend conferences in exotic locations than in attending society-sponsored conferences in the usual conference-hosting cities [3].

Specialty societies historically have responded better to research challenges. Declining federal funding, bureaucratic complexity, and inequities in research opportunities necessitate that medical societies serve as advocates and incubators for young investigators [10].

A major area of potential value, accessed by very few organizations, is developing centers for research activities. Some societies have taken steps to establish data centers and repositories for real-world evidence that can serve as platforms for members to conduct research using tools derived from artificial intelligence and natural language processing to acquire both unstructured and structured data in diseases within the societies’ domains. Funding these initiatives is challenging, but a national society may have a greater likelihood of successfully curating data from member organizations than an ad hoc collection of academic institutions. The potential service of such repositories to the membership and to patients is large, serving both research and patient-care goals [9].

Developing and encouraging physician leaders may be the greatest challenge to the vitality and relevance of medical societies. While many organizations have given attention to fostering inclusivity and diversity, ensuring that societies reflect both the geographic and political diversity of physicians and the patients that they serve will mean that organizations will need to draw more broadly from the communities they represent. Many of our national societies remain invisible to patient-advocacy groups and, indeed, the lay public at large. This has the unfortunate effect of making specialty societies appear elitist and irrelevant to patients and philanthropists [11]. A way forward would be for medical societies to develop community advisory boards with which they could partner to help focus their activities.

Recommendations for the future

To remain vital and relevant, medical societies must evolve to foster inclusivity of diverse groups in the profession as well as working productively with the diverse groups that compose the lay public. Societies would be well-advised to embrace digital resources that expand virtual conferences and broaden access, thus reducing barriers to participation. Supported research that strengthens interdisciplinary collaboration and facilitates partnerships between specialties, subspecialties, and allied health professionals to address complex healthcare challenges will lead to increased relevance and legitimacy.

For the physician members, leadership should evolve from the rotation of those with a long history of involvement, nominated through arcane processes, and elected by a minority of members who have the time to navigate a process skewed toward those with truncated clinical and/or scientific accomplishments [12]. Novel ways of presenting slates to most members extended over longer intervals, with tangible benefits for engagement, may help democratize governance. Societies that broaden the scope of leadership with training and outreach and that seek to involve members with a legitimate history of community involvement and education stand to accomplish the most.

Medical societies will need to demonstrate tangible impact on members’ careers, education, and well-being, making membership indispensable (see Table 1). They will need to develop structured mentorship and leadership training programs that empower early-career professionals and use advocacy to ensure that the society remains a leader in calling for research funding, patient-centered policies, and equitable healthcare delivery [11,13]. Moreover, societies will need to demonstrate their relevancy to young professionals who perhaps view medicine more as a profession than a calling and who perceive all their professional needs being met by their employer. For example, if medical societies gathered and shared more specific knowledge of and research on large regional medical groups (by name), comparing benefits, salaries, professional autonomy, institutional support, and work-life balance, such proprietary information would be of great interest to member physicians in training as well as early-career physicians. Moreover, such a database might encourage employers to “up their game” to remain competitive.

**Table 1:** Interventions to increase the value of academic medical societies.

Domain	Intervention	Expected impact
Membership value	Employer comparison databases	Improved transparency
Engagement	Virtual/hybrid meetings	Increased participation
Leadership	Transparent pathways	Broader inclusion
Research	Centralized registries	Enhanced research output
	Awards and bridge grants	
Advocacy	Reduce administrative burden	Lower burnout
	Symposia on AI serves within the EMR	
Community	Convene patient advisory boards	Improved trust

Conclusion

Medical societies have been integral to the professionalization of medicine in the United States and continue to play vital roles in advancing science, education, and advocacy. Yet in an era of rapid change and increasing complexity, societies must adapt to remain relevant and develop metrics, including survey instruments, publication and political action indicators, and artificial intelligence-driven tools to measure the success of each novel intervention. By embracing inclusivity, digital innovation, leadership cultivation, and strategic advocacy, they can continue to support the next generation of practitioners, researchers, educators, and clinical investigators, ensuring value for the profession in the decades ahead.
